# Development and Validation of Predictive Assessment of Complicated Diverticulitis Score

**DOI:** 10.3390/jpm11020080

**Published:** 2021-01-29

**Authors:** Marcello Covino, Valerio Papa, Antonio Tursi, Benedetta Simeoni, Loris Riccardo Lopetuso, Lorenzo Maria Vetrone, Francesco Franceschi, Gianludovico Rapaccini, Antonio Gasbarrini, Alfredo Papa

**Affiliations:** 1 Emergency Medicine, Fondazione Policlinico Universitario A. Gemelli, IRCCS, 00168 Rome, Italy; macovino@gmail.com (M.C.); benedetta.simeoni@policlinicogemelli.it (B.S.); francesco.franceschi@policlinicogemelli.it (F.F.); 2 Facoltà di Medicina e Chirurgia, Università Cattolica del S. Cuore, L.go A. Gemelli 8, 00168 Rome, Italy; valerio.papa@policlinicogemelli.it (V.P.); vetrone.md@gmail.com (L.M.V.); gianludovico.rapaccini@policlinicogemelli.it (G.R.); antonio.gasbarrini@policlinicogemelli.it (A.G.); 3 Digestive Surgery, Fondazione Policlinico Universitario A. Gemelli, IRCCS, 00168 Rome, Italy; 4 Territorial Gastroenterology Service, Azienda Sanitaria Locale Barletta-Andria-Trani, 70031 Andria, Italy; antotursi@tiscali.it; 5 Department of Medicine and Ageing Sciences, G. d’Annunzio University of Chieti-Pescara, 66100 Chieti, Italy; lorislopetuso@libero.it; 6 Center for Advanced Studies and Technology (CAST), G. d’Annunzio University of Chieti-Pescara, 66100 Chieti, Italy; 7 Internal Medicine and Gastroenterology, Fondazione Policlinico Universitario A. Gemelli, IRCCS, 00168 Rome, Italy

**Keywords:** acute diverticulitis, complicated diverticulitis, prognostic score, surgery, abscess, perforation, diverticular hemorrhage

## Abstract

The prevalence of acute diverticulitis (AD) has progressively increased in recent decades, with correspondingly greater morbidity and mortality. The aim of the study is to develop a predictive score to identify patients with the highest risk of complicated AD. The clinical records of 1089 patients referred to the emergency department (ED) over a five-year period were reviewed. In multivariate analysis, male sex (*p* < 0.001), constipation (*p* = 0.002), hemoglobin < 11.9 g/dL (*p* < 0.001), C reactive protein > 80 mg/L (*p* < 0.001), severe obesity (*p* = 0.049), and no proton pump inhibitor treatment (*p* = 0.003) were independently associated with complicated AD. The predictive assessment of complicated (PACO)-diverticulitis (D) score, including these six variables, was applied to the retrospective cohort and then validated prospectively in a cohort including 282 patients. It categorized patients into three risk classes for complicated AD. The PACO-D score showed fair discrimination for complicated AD with an area under the receiver operating characteristic curve of 0.674 and 0.648, in the retrospective and prospective cohorts, respectively. The PACO-D score could be a practical clinical tool to identify patients at highest risk for complicated AD referred to the ED so that appropriate diagnostic and therapeutic resources could be appropriately allocated. Further external validation is needed to confirm these results.

## 1. Introduction

Acute diverticulitis (AD) and its complications are the most difficult-to-manage clinical manifestations of diverticular disease of the colon [1]. The clinical picture of AD is extremely variable in its severity, ranging from mildly symptomatic forms that resolve rapidly to life-threatening conditions due to the onset of complications requiring emergency surgery [2]. Indeed, complicated AD, as defined by the presence of any of the following manifestations: abscess, peritonitis, obstruction, fistula, or hemorrhage, is burdened with considerable morbidity and mortality [3]. In the last two decades, particularly in Western countries, the prevalence and hospitalization rate of AD and its complications have progressively increased, placing a considerable burden on national health systems [4,5,6]. As expected, most of the costs are attributed to hospitalizations, especially for emergency admissions and any subsequent surgery [7,8]. On the other hand, in most cases, uncomplicated AD is relatively mild and often self-limiting and can be safely managed in an outpatient setting with significant savings on healthcare resource costs [9,10]. Therefore, early identification of patients at the highest risk of complicated AD would be of great benefit, particularly for emergency department (ED) physicians. Indeed, having a reliable diagnostic tool capable of predicting the severity of AD at the initial presentation and estimating the outcome of these patients would make ED physicians more confident in focusing on the diagnostic and therapeutic efforts in complicated cases and, consequently, in the management of mild cases on an outpatient basis. Thus, the aim of this study was to develop and validate an easy-to-apply score, which includes several variables independently associated with complicated AD, in order to identify the patients at highest risk of developing complicated AD among all patients referred to the ED with a diagnosis of AD.

## 2. Results

### 2.1. Demographic Data of the Derivation Cohort

During the retrospective study period, 1343 patients with AD were evaluated in our ED. Two hundred and fifty-four patients were excluded for insufficient or inconsistent clinical data. Finally, 1089 patients were included in the study cohort: 46% were male and the overall median age was 66 (IQR, 53–77) years. The number of patients with complicated AD was 296 (27.2%). The complications of AD were as follows: perforation in 110 (10.1%), diffuse peritonitis in 32 (2.9%), abdominal/pelvic phlegmon or abscess in 53 (4.9%), diverticular bleeding in 200 (18.4%), intestinal fistulas in 8 (0.7%), and stenosis in 4 (0.3%) patients. Some patients had more than one complication.

### 2.2. Parameters Associated with Complicated AD in Univariate Analysis

The univariate model analysis indicated that several parameters were significantly associated with the development of complicated AD (Table 1). Male sex (*p* = 0.001), abdominal pain (*p* = 0.006), constipation (*p* = 0.029), hypertension (*p* = 0.002), obesity (*p* = 0.019), and heavy active smoking (*p* = 0.006) were risk factors for complicated AD. Conversely, PPI (*p* = 0.001) consumption was associated with a protective effect at the onset of complicated AD. In the univariate analysis, all the investigated markers of inflammation or anemia were significantly associated with complicated AD: CRP (*p >* 0.001), WBC (*p* = 0.009), and fibrinogen (*p* = 0.001), as well as decreased levels of Hb (*p >* 0.001).

### 2.3. Parameters Associated with Complicated AD in Multivariate Analysis

In the multivariate logistic regression analysis for factors associated with complicated AD, male sex (OR 1.73, 95% CI 1.29–2.32, *p* < 0.001), constipation (OR 1.95, 95% CI 1.26–2.91, *p* = 0.002), Hb levels < 11.9 g/dL (OR 2.40, 95% CI 1.74–3.32, *p* < 0.001), CRP *>* 80 mg/mL (OR 1.84, 95% CI 1.32–2.57, *p* < 0.001), obesity (OR 1.022, 95% CI 0.92–0.95, *p* < 0.0001), and not being on PPI (OR 3.94, 95% CI 2.26–6.86, *p* < 0.0001) remained significantly correlated with complicated AD (Table 2).

### 2.4. Outcomes

As expected, in-hospital mortality (*p* = 0.001), major complications (*p* < 0.001), surgery (*p* < 0.001), percutaneous drainage (*p* < 0.001), and colostomy placement (*p* < 0.001) were significantly more frequent in patients with complicated AD. LOS was significantly lower in patients with uncomplicated AD (*p* < 0.001) (Table 3).

### 2.5. The PACO-D Score Development and Application in the Derivation Cohort

**Figure 1:**
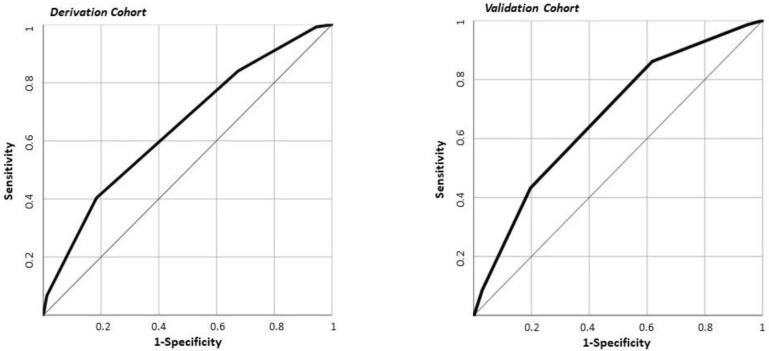
Receiver operating characteristic (ROC) curve of PACO-D score with respect to complicated diverticulitis in derivation (1089 patients) and in validation (282 patients) cohorts. The area under the ROC curve was 0.674 (0.638–0.709) in the derivation cohort and 0.648 (0.584–0.713) in the validation cohort (*p* = 0.489).

The variables included in the PACO-D score were as follows: male sex, constipation, obesity (BMI > 30), not being on PPI therapy, Hb levels < 11.9 g/dL, and CRP *>* 80 mg/dL. In the derivation cohort, 11.9% of patients with complicated AD were in the low-risk class, 33% in the moderate-risk class, and 52.1% in the high-risk class according to the PACO-D score (Table 4). In the derivation cohort, the area under the ROC curve for the PACO-D score with respect to complicated AD was 0.674 (0.638–0.709) (Figure 1). Compared to low-risk patients, moderate-risk patients had an OR of 3.6 (2.5–5.2) for complicated AD, whereas patients in the high-risk group had an OR of 8 (4.2–15.4).

### 2.6. Demographic, Clinical Data, and Outcomes of the Patients Included in the Validation Cohort

The demographic and clinical features of the patients included in the validation cohort (282 patients, 119 with complicated AD) are reported in Supplementary Table S1. The complications of AD were as follows: perforation in 52 (18.4%), diffuse peritonitis in 10 (3.5%), abdominal/pelvic phlegmon or abscess in 21 (7.4%), diverticular bleeding in 65 (23%), and fistulas in 2 (0.7%) patients. Some patients had more than one complication. Their outcomes are shown in Supplementary Table S2.

### 2.7. The PACO-D Score Application in the Validation Cohort

In the validation cohort, 22.4% of patients with complicated AD were in the low-risk class, 42.4% in the moderate-risk class, and 60.5% in the high-risk class according to the PACO-D score (Table 4). In the validation cohort, the area under the ROC curve for the PACO-D score with respect to complicated AD was 0.648 (0.584–0.713). This result was not statistically different than the area under the ROC curve obtained in the derivation cohort (*p* = 0.489) (Figure 1). Compared to the patients at low risk, patients with moderate risk had an OR of 2.5 (1.4–4.9) for complicated AD, whereas patients in the high-risk group had an OR of 5.3 (2.6–10.7).

### 2.8. The PACO-D Score and Cumulative Major Complications

In the derivation cohort, 1.2% of patients with cumulative major complications were in the low-risk class, 2.6% in the moderate-risk class, and 6.3% in the high-risk class according to the PACO-D score. In the validation cohort, 1.3% of patients with cumulative major complications were in the low-risk class, 3.2% in the moderate-risk class, and 9.9% in the high-risk class (Table 4). The area under the ROC curve was 0.603 (0.494–0.712) in the derivation cohort and 0.698 (0.558–0.837) in the validation cohort (*p* = 0.293).

## 3. Discussion

In the last decade, the average age of the population, particularly in industrialized countries, has been rising and in parallel, the absolute number of ED attendances by the elderly has also been increasing [11]. A significant number of these accesses to the ED among non-neoplastic digestive diseases is due to AD since it is a very common condition whose prevalence increases with age [12]. Consequently, AD represents a substantial burden on health systems worldwide, mainly attributable to emergency hospitalizations and surgeries [5,6,7,8]. Indeed, complications occur in approximately 12% of patients with AD, and mortality after complicated diverticulitis is highest among individuals with perforation or abscesses [13,14]. In our study, we also included patients with diverticular bleeding among the complicated cases since it is a common cause of lower gastrointestinal hemorrhage in adults, sometimes requiring surgery or arterial embolization after the failure of endoscopic hemostasis [15,16]. Recently, in a cohort of 99 patients with documented diverticular bleeding, 23 had a severe hemorrhage, of which 7 required emergency surgeries [17]. Thus, it is extremely important to identify patients with AD who are at the highest risk of having a complicated disease and, consequently, a worse outcome.

Several studies have been conducted to identify a single risk factor or a score that includes multiple variables to confirm the diagnosis of AD in patients with this clinical suspicion, but none have been shown to have significant reliability [18,19]. Therefore, to date, a full evaluation of patients using clinical history, signs, and laboratory inflammation markers is recommended before performing a contrast-enhanced CT scan of the abdomen, which is considered the first-choice imaging method to obtain a definitive diagnosis of AD [20]. However, once the diagnosis of AD has been established, it is equally important to define the presence of complicated or uncomplicated AD in order to implement proper patient management (medical or surgical treatment) and the setting of care (hospital or outpatient) [9,10,20,21]. A contrast-enhanced CT scan allows for the identification of the presence of complicated AD, according to the most common classification available [21,22], and sometimes it may even be able to diagnose the presence of active diverticular bleeding before patients with significant rectal bleeding undergo colonoscopy [23]. Nevertheless, it would be extremely helpful for the treating ED physicians to have an easy-to-apply score that can provide a pre-test probability for complicated AD at the time of the CT scan. Indeed, stratifying the individual risk for a more severe course of AD as soon as possible is crucial for patient management because it would allow diagnostic and therapeutic efforts to be directed towards patients considered to be at greatest risk. Recently, two systematic reviews were carried out to identify significant factors that could predict complicated AD [24,25]. However, these risk factors, when individually considered, showed unsatisfactory discriminative value towards complicated AD. Thus, Bolkenstein et al. proposed a prognostic model combining the most significant risk factors obtained from the literature data [25]. This model is neither developed nor validated in any patient cohort yet. For all these reasons, we have developed and validated a predictive score, the PACO-D score, aiming to aid the decision-making process in the event of a suspected complicated AD. Indeed, it ranks patients into three risk classes for complicated AD: patients of the derivation cohort in the high-risk group showed an eight-fold greater risk of complicated AD and a six-fold greater risk of cumulative major complications (including admission to the intensive care unit/mechanical ventilation, sepsis, or death) than those in the low-risk group considered as reference. A strength of this score is that it can be applied immediately to the patient’s bedside because it includes six items routinely evaluated in patients who are referred to ED for acute abdominal symptoms. Four of these are clinical variables derived from history (male sex, severe obesity, presence of constipation, and not being on PPI therapy) and two are laboratory values (Hb and CRP). Furthermore, as these parameters could be collected in any ED without additional time or cost, we believe that the use of the PACO-D score could be of great help in the management of patients with suspected complicated AD even in non-referral hospitals.

In a retrospective study, Longstreth et al. identified constipation and male sex as two independent risk factors for severe AD, defined by a modified Hinchey classification from stage IB to stage IV as evidenced by CT scan [26]. In addition, fever > 37.5 °C and leucocytes > 11,000/mm^3^ were independently associated with severe AD, although only 25% of the patients included in their cohort with severe AD had contemporary fever and leukocytosis [26]. Among the risk factors included in the PACO-D score, two deserve further comments. The first one is the CRP value. In fact, several studies have reported CRP as an independent risk factor for complicated AD [27,28,29,30]. The optimal threshold value to distinguish uncomplicated from complicated AD ranged in different studies from 90 mg/L [27] to 149.5 mg/L [28] up to 175 mg/L [29]. Furthermore, data obtained in a cohort of patients with AD showed that the perforation was unlikely when CRP was < 50 mg/L, whereas only values > 200 mg/L were a strong indicator of perforation [30]. The difference in the discriminant cut-off value of CRP lies in the heterogeneity of the populations considered, and although the specificity for complicated AD increases as the CRP value increases, the sensitivity becomes progressively more disappointing. In our patient cohorts, we confirmed CRP as an independent risk factor for complicated AD, and a lower cut-off (CRP *>* 80 mg/L) was selected for the PACO-diverticulitis score in order to increase its predictability when combined with the other selected items of the score. A second factor included in the score to be commented on is the role of PPIs. Among the medications considered as a risk or protective factor for complicated AD, PPIs are of interest because they are a relatively less investigated class of drugs despite their widespread use in the population. The studies published so far on the role of PPIs in AD and their complications have shown partially conflicting results [31,32]. In the first study, Ho et al. reported that the use of PPIs did not increase the risk of diverticulitis [31], whereas in the second study, Sbeit et al. showed that PPI use was significantly associated with diverticulitis, but did not affect its severity [32]. Accordingly, in the present study, we found that being on PPI therapy was associated with a reduced risk of complicated AD. In addition, Tursi et al. recently reported that PPI use was not significantly associated with the endoscopic severity of diverticular disease, according to the Diverticular Inflammation and Complications Assessment (DICA) classification [33]. PPI therapy was associated with a decrease in α diversity and taxonomic changes in the gut microbiota, including a decrease in *Clostridiales* and an increase in *Actinomycetales, Micrococcaceae*, and *Streptococcaceae*, which are implicated in dysbiosis and increased susceptibility to *Clostridioides difficile* infection [34]. These PPI-induced modifications of the gut microbiota could at best favor the onset of AD but not its complications. At present, this hypothesis is purely speculative, as there are no experimental data to support it. However, these findings could also provide a further rationale for the use of different probiotics depending on the stage of diverticular disease [35].

## 4. Materials and Methods

### 4.1. Study Design, Patient Enrollment, and Selection

This was a five-year retrospective study followed by a one-year prospective study conducted in a teaching hospital with annual attendance at the ED of about 75,000 patients (more than 87% adults). All clinical records of patients admitted to the ED from 1 January 2014 to 31 December 2018 were evaluated, and patients with a diagnosis of AD were included in the retrospective study (derivation cohort). A prospective study was then started that included all consecutive patients referred to the ED from 1 January 2019 to 31 December 2019 with a diagnosis of AD (validation cohort). In detail, for the patients in the derivation cohort, we extracted information collected on the admission of patients with a diagnosis of AD of the colon (International Classification of Disease, 9th Revision, Clinical Modification (ICD-9-CM), ICD-9-CM code 562.11 (diverticulitis without mention of hemorrhage), and 562.13 (diverticulitis with mention of hemorrhage)) either as a primary diagnosis or as a secondary diagnosis but with a complication of diverticulitis as the primary diagnosis. We excluded patients under 18 years of age, pregnant women, and patients with a diagnosis of colon or rectal cancer (ICD-9-CM 153.0–153.9, 197.5). AD diagnosis was suspected clinically and with the aid of the patients’ clinical history and then confirmed by a computed tomography (CT) scan of the abdomen. CT scans also allowed us to distinguish patients with complicated AD from those with uncomplicated AD according to the modified Hinchey classification [22]. In detail, uncomplicated AD was defined as the Hinchey 0 or Ia stages (mild clinical diverticulitis or colonic wall thickening and/or confined pericolic inflammation) and complicated AD as Ib stage (confined small pericolic abscess, ≤5 cm) and II–IV stages (pelvic, distant intra-abdominal, or retroperitoneal abscess; generalized purulent or fecal peritonitis, fistula, and obstruction) [22]. Furthermore, patients with AD associated with rectal bleeding were included among patients with complicated AD if a CT scan and/or a successive colonoscopy performed during hospitalization showed active bleeding from diverticula or stigmata from recent diverticular bleeding (nonbleeding visible vessel or an adherent clot) without any other evident sources of bleeding [15,23]. The study was conducted in accordance with the Declaration of Helsinki and approved by the Local Ethics Committee for the research purpose use of the data, stemming from standard clinical practice since no additional interventions were planned (observational study). All patients provided informed consent to participate in the study.

### 4.2. Data Collection

Demographic variables assessed included age, sex, and current heavy smoking, defined as cigarette consumption of more than one pack per day. At admission, clinical data recorded included the chief complaints (fever, abdominal pain, diarrhea, vomiting, constipation, weight loss, and rectal bleeding) and the comorbidities included in the Charlson comorbidity index [36]. Previous episodes of AD and obesity, defined as a body mass index (BMI) ≥ 30, were also registered. Data were recorded on the most common medications that could have an impact on the course of AD, in particular aspirin (taken at least every day in the previous month), non-steroidal anti-inflammatory drugs (NSAIDs) (taken at least every day in the previous week), oral anticoagulants (taken for at least one month), steroids (every dosage taken for at least one month in the last 6 months), proton pump inhibitors (PPIs) (taken every day for at least one month), and statins (taken every day for at least one month). Laboratory predictors at admission included hemoglobin (Hb), white blood cells (WBC), C-reactive protein (CRP), and fibrinogen.

### 4.3. Outcomes

The outcomes assessed for patients with complicated and uncomplicated AD were in-hospital mortality, major surgery, any surgical procedure (including major surgery and minor surgery/invasive radiological procedure), sepsis, major complications (including at least one of the following: admission to intensive care unit/mechanical ventilation, sepsis, or death), colostomy placement, and length of hospital stay (LOS), calculated from ED admission to discharge/death. In detail, we considered percutaneous drainage or laparoscopic peritoneal lavage and drainage as a “minor surgery/invasive radiological procedure.” “Major surgery” included a colonic resection, either Hartmann’s procedure or a resection with primary anastomosis and colostomy.

### 4.4. Statistical Analysis

Continuous variables are presented as median (interquartile range (IQR)) and compared using the Mann–Whitney U test. Categorical variables are presented as A number (percentage) and compared using the Chi-squared test or Fisher’s exact test, as appropriate. A two-sided *p*-value of 0.05 or less was regarded as significant. Study variables significantly associated with complicated AD in univariate analysis were entered in a multivariate logistic regression model in order to identify independent predictors of complicated AD. Prior to entering the logistic regression model, continuous variables were dichotomized using receiver operating characteristic (ROC) analysis. For each variable, a cut-off based on a sensitivity value ≥ 80% was selected for association with complicated diverticulitis. Sensitivity, specificity, and THE area under the ROC curve are presented as values (95% confidence interval (CI)). Logistic regression results are presented as odds ratios (ORs) (95% CI). To reduce redundancy in the logistic regression model, clinical history factors already included in the Charlson comorbidity index were excluded from the models. The goodness of fit of our model was assessed using the Hosmer–Lemeshow test.

### 4.5. Development of the Predictive Score

We aimed to develop a simple score that could accurately predict the risk for complicated AD at patients’ bedside evaluations in the ED. The variables identified as independently associated with complicated AD at multivariate regression analysis in the derivation cohort were included in the new score. Based on standardized coefficients in the linear model, we found that the incremental contribution of each variable to association with complicated AD was similar (about +10% each). Hence, each variable was given a + 1 value to create the PACO-D score, ranging from 0 to 6 points. Based on the derived score, we visually evaluated the calibration value of the score in the derivation and the validation cohorts, and then assigned a low risk for values 0–1, medium risk for values 2–3, and high risk for values ≥ 4. Score performance was evaluated using the area under the ROC curve (95% CI) with respect to the presence of complicated AD, and with respect to the occurrence of cumulative major complications.

### 4.6. Score Validation

We validated the PACO-D score in a prospective cohort of patients accessing the ED for AD. Score discrimination ability for complicated AD and the occurrence of cumulative major complications were assessed using the area under the ROC curve (95% CI). The comparison of the PACO-D score performance between the validation and derivation cohorts was assessed with the DeLong method.

### 4.7. Sample Size

Since 12 variables were entered into the logistic regression model, a total of 120 complicated ADs would have been required in the study cohort for a satisfactory parameter estimation. The derivation cohort largely outnumbered these figures. At the same time, since approximately 1/3 of patients had complicated AD, and the final proposed score had 6 variables, at least 180 patients would have been required for validation. Again, the study validation population was sufficient for the analysis. All data were analyzed using SPSS v25^®^ (IBM, Armonk, NY, USA).

## 5. Conclusions

There are some limitations of this study to consider when interpreting the results. First, this is a monocentric study and, consequently, the proposed score should be further validated in other external cohorts. Second, some epidemiological risk factors involved in the occurrence of complicated AD, such as dietary habits (dietary fiber consumption, vegetarian diet, red meat intake, alcohol assumption), physical activity, and insolation were not included in our analysis. Finally, we underline that the PACO-D score should be considered as a supportive tool to the CT scan of the abdomen, which remains essential not only to confirm the diagnosis of complicated AD but above all to plan the subsequent treatment, be it surgical, radiologically guided abscess drainage, or medical. On the other hand, the PACO-D score, developed in a large sample of patients with AD and then validated in an equally large prospective cohort, may represent a useful prognostic model in the management of patients with AD referred to the ED. Indeed, it can help physicians to quickly identify patients at high risk of complicated AD, so that they can be alerted to a poorer prognosis and, consequently, the patients can be triaged and managed accordingly.

## Figures and Tables

**Table 1:** Comparison of demographic and clinical characteristics between patients with complicated and uncomplicated diverticulitis at emergency department admission.

Variable	All Population *n* = 1089	Uncomplicated Diverticulitis *n* = 793	Complicated Diverticulitis *n* = 296	*p*-Value
Age (Years), median (IQR)	66 (53–77)	65 (53–77)	67 (54–79)	0.106
Sex (Male), n (%)	501 (46.0)	341 (43.0)	160 (54.1)	0.001
Presentation, n (%)				
Fever	429 (39.4)	324 (40.9)	105 (35.5)	0.106
Abdominal pain	753 (69.1)	567 (71.5)	186 (62.8)	0.006
Vomit	205 (18.8)	153 (19.3)	52 (17.6)	0.517
Constipation	144 (10.5)	89 (9.3)	55 (13.3)	0.029
Diarrhea	151 (13.9)	111 (14.0)	40 (13.5)	0.837
Weight loss	27 (2.5)	20 (2.5)	7 (2.4)	0.882
Therapy, n (%)				
PPIs	135 (12.4)	114 (14.4)	21 (7.1)	0.001
Aspirin	151 (13.9)	117 (14.8)	34 (11.5)	0.165
NSAIDs in previous week	50 (4.6)	35 (4.4)	15 (5)	0.646
Steroids	49 (4.5)	35 (4.4)	14 (4.7)	0.823
Anticoagulation (VKA)	46 (4.2)	34 (4.3)	12 (4.1)	0.865
Statin	62 (5.7)	51 (6.4)	11 (3.7)	0.085
Laboratory Values, Median (IQR)				
Hemoglobin (g/dL)	13.3 (11.9–14.5)	13.5 (12.3–14.6)	12.8 (10.9–14.1)	<0.001
WBC (×10^9^/L)	9.0 (6.2–12.0)	9.0 (6.1–12.0)	10.1 (6.5–13.6)	0.009
Fibrinogen (mg/dL)	459 (341–604)	446 (335–582)	497 (362–642)	0.001
C-reactive protein (mg/L)	46 (23–80)	45 (21–74)	48 (26–106)	<0.001
Comorbidities				
Charlson comorbidity index, median (IQR)	2 (1–4)	2 (1–4)	2 (1–4)	0.426
First episode of AD, n (%)	773 (71.0)	547 (69.0)	226 (76.4)	0.017
Hypertension, n (%)	149 (13.7)	93 (11.7)	56 (18.9)	0.002
Obesity, n (%)	10 (0.9)	4 (0.5)	6 (2.0)	0.019
Heavy smoker, n (%)	73 (6.7)	43 (5.4)	30 (10.1)	0.006

Abbreviations: IQR, interquartile range; VKA, vitamin K antagonists; PPIs, Proton Pump Inhibitors; NSAIDs, non-steroidal anti-inflammatory drugs; WBC white blood count.

**Table 2:** Multivariate analysis (logistic regression) for factors associated with complicated diverticulitis.

Factor	ß	SE	OR (95% CI)	*p*-Value
Sex (Male)	0.548	0.150	1.73 (1.29–2.32)	<0.001
Abdominal Pain	−0.228	0.164	0.79 (0.58–1.09)	0.163
Constipation	0.650	0.214	1.95 (1.26–2.91)	0.002
Not on PPIs	0.766	0.262	2.15 (1.29–3.59)	0.003
Hemoglobin < 11.9 (g/dL)	0.876	0.165	2.40 (1.74–3.32)	<0.001
WBC > 12.9 (×10^9^/L)	0.147	0.177	1.16 (0.82–1.64)	0.407
Fibrinogen > 623 (mg/dL)	0.171	0.177	1.16 (0.84–1.68)	0.334
C-reactive protein > 80 (mg/L)	0.612	0.169	1.84 (1.32–2.57)	<0.001
First episode of AD	0.221	0.173	1.25 (0.88–1.75)	0.203
Hypertension	0.310	0.216	1.36 (0.89–2.08)	0.151
Obesity	1.350	0.688	3.86 (1.01–14.85)	0.049
Heavy smoker	0.367	0.282	1.44 (0.83–2.51)	0.194

Model Chi^2^ = 110.2, log likelihood = 1164.029; goodness of fit (Hosmer and Lemeshow) *p* = 0.111. Constant was included in the model. Abbreviations: SE, standard error; OR, odds ratio; CI, confidence interval; PPIs, proton pump inhibitors; WBC white blood count; AD, acute diverticulitis.

**Table 3:** Comparison of patient outcomes between complicated and uncomplicated diverticulitis.

Variable	All Population *n* = 1089	Uncomplicated Diverticulitis *n* = 793	Complicated Diverticulitis *n* = 296	*p*-Value
Death, n (%)	11 (1.0)	3 (0.4)	8 (2.7)	0.001
Sepsis, n (%)	14 (1.3)	7 (0.9)	7 (2.4)	0.053
Mechanical ventilation, n (%)	9 (0.8)	0	9 (3.0)	<0.001
Major complications ^†^, n (%)	25 (2.3)	10 (1.3)	15 (5.1)	<0.001
Any surgical procedure, n (%)	95 (8.7)	11 (1.4)	84 (28.4)	<0.001
Major surgery, n (%)	82 (7.5)	7 (0.9)	75 (25.3)	<0.001
Percutaneous drainage, n (%)	13 (1.2)	4 (0.5)	9 (3.0)	<0.001
Colostomy, n (%)	33 (3.0)	2 (0.3)	31 (10.5)	<0.001
Total LOS ^‡^, median (interquartile range)	2.7 (0.3–5.8)	0.8 (0.3–3.8)	6.9 (4.4–10.3)	<0.001

Results are expressed as number (percentage) or median (interquartile range) as appropriate. Abbreviations: LOS, length of hospital stay. ^†^ Major complications include admission to intensive care units/mechanical ventilation, sepsis, or death. ^‡^ LOS is calculated from emergency department admission to hospital discharge.

**Table 4:** Summary of the proposed PACO-D score on derivation cohort (4-A) and on validation cohort (4-B).

**4-A Derivation Cohort (1089 Patients)**					
**PACO Risk Class**	**Score** **Values**	**Complicated Diverticulitis** **n (%)**	**Relative Risk** **OR (95% CI)**	**Cumulative Major Complications** **n (%)**	**Relative Risk** **OR (95% CI)**
Low Risk	0–1	41 (11.9)	*Reference*	4 (1.2)	*Reference*
Moderate Risk	2–3	230 (33.0)	3.6 (2.5–5.2)	18 (2.6)	2.2 (0.7–6.7)
High Risk	≥4	25 (52.1)	8.0 (4.2–5.4)	3 (6.3)	5.7 (1.2–26.1)
**4-B Validation Cohort (282 Patients)**					
**PACO Risk Class**	**Score** **Values**	**Complicated** **Diverticulitis** **n (%)**	**Relative Risk** **OR (95% CI)**	**Cumulative Major Complications** **n (%)**	**Relative Risk** **OR (95% CI)**
Low Risk	0–1	17 (22.4)	*Reference*	1 (1.3)	*Reference*
Moderate Risk	2–3	53 (42.4)	2.4 (1.4–4.9)	4 (3.2)	2.5 (0.3–22.6)
High Risk	≥4	49 (60.5)	5.3 (2.6–0.7)	8 (9.9)	8.2 (1.1–7.4)

PACO-D score consists of +1 values for each of the following items: male sex, severe obesity (BMI > 30), constipation, not on PPI therapy, hemoglobin < 11.9 g/dL, C-reactive protein > 80 mg/L. Abbreviations: OR, odds ratio; CI, confidence interval.

## Data Availability

The data presented in this study are available on request from the corresponding author. The data are not publicly available due to privacy restrictions.

## Supplementary Materials

The following are available online at https://www.mdpi.com/2075-4426/11/2/80/s1, Table S1: Comparison of demographic and clinical characteristics between patients with complicated and uncomplicated diverticulitis at emergency department admission (validation cohort); Table S2: Validation cohort: comparison of patient outcomes between complicated and uncomplicated diverticulitis.
