# Supplementary material for: Development and Validation of Predictive Assessment of Complicated Diverticulitis Score
Source: J Pers Med. 2021 Jan 29;11(2):80. doi: 10.3390/jpm11020080 (PMC7911244; doi:10.3390/jpm11020080)
Supplement: Supplementary file 1 [file jpm-11-00080-s001.pdf]

Supplementary Figure 1. Receiver Operating Characteristic (ROC) curve of PACO-D score with respect to cumulative major endpoint (death, admission to intensive care unit/Ventilation, sepsis) in derivation (1089 patients) and in validation (282 patients) cohorts. Area under ROC curve was 0.603 (0.494 – 0.712) in derivation cohort and 0.698 (0.558 – 0.837) in validation cohort (p=0.293).

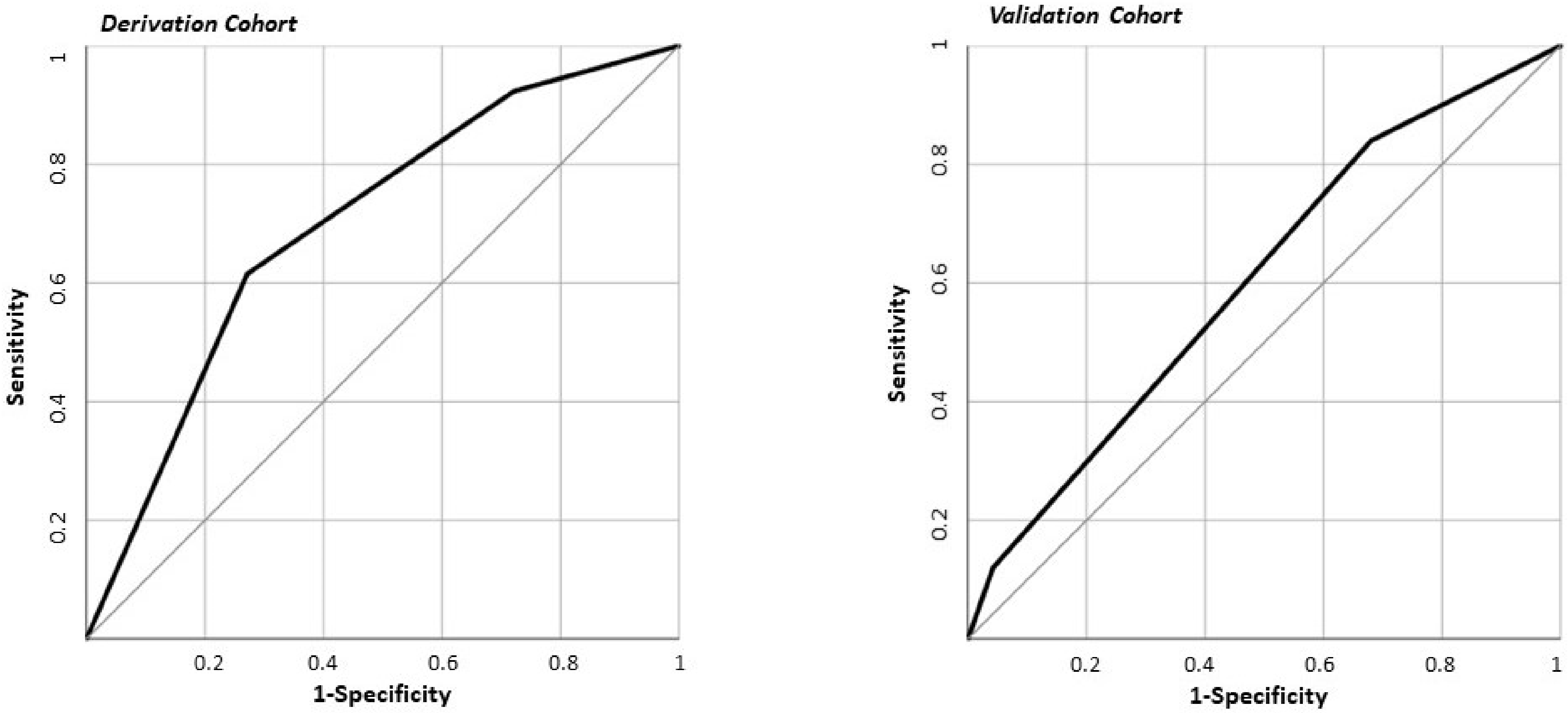

Supplementary Table 1 –Comparison of demographic and clinical characteristics between patients with complicated and uncomplicated diverticulitis at emergency department admission (validation cohort).

**Variable**	**All population n 282**	**Uncomplicated diverticulitis n 163**	**Complicated diverticulitis n 119**	**p**
Age (Years), median (IQR)	69 (57 – 81)	68 (56 – 78)	73 (57 – 84)	0.102
Sex (Male)	129 (45.7)	71 (43.6)	58 (48.7)	0.388
Presentation				
Fever	113 (40.1)	70 (42.9)	43 (36.1)	0.249
Abdominal pain	197 (69.9)	118 (72.4)	79 (66.4)	0.278
Vomit	54 (19.1)	34 (20.9)	20 (16.8)	0.393
Constipation	16 (5.7)	7 (4.3)	9 (7.6)	0.241
Diarrhea	44 (15.6)	31 (19.0)	13 (10.9)	0.064
Weight loss	13 (4.6)	12 (7.4)	1 (0.8)	0.009
Therapy				
PPIs	36 (12.8)	29 (17.8)	7 (5.9)	0.003
Aspirin	60 (21.3)	44 (27.0)	16 (13.4)	0.006
FANS in previous week	13 (4.6)	11 (6.7)	2 (1.7)	0.045
Steroid	13 (4.6)	7 (4.3)	6 (5.0)	0.767
Anticoagulation (VKA)	9 (3.2)	3 (1.8)	6 (5.0)	0.131
Statin	17 (6.0)	12 (7.4)	5 (4.2)	0.271
Laboratory Values				
Hemoglobin (g/dL)	12.9 [11.2 – 14.2]	13.1 [11.4 – 14.3]	12.5 [10.4 – 14.1]	0.015
WBC (x10^9/L)	9.0 [6.0 – 12.0]	8.7 [6.0 – 12.1]	9.1 [6.5 – 14.5]	0.006
Fibrinogen (mg/dL)	474 [364 – 611]	474 [359 – 607]	476 [373 – 622]	0.711
C reactive protein (mg/L)	47 [21 – 82]	41 [16 – 71]	55 [34 – 105]	<0.001
Comorbidities				

Charlson Comorbidity index	2 [1 – 4]	2 [1 – 4]	2 [1 – 4]	0.637
First episode diverticulitis	208 (73.8)	116 (71.2)	92 (77.3)	0.247
Hypertension	35 (12.4)	17 (10.4)	18 (15.)	0.237
Obesity	8 (2.8)	3 (1.8)	5 (4.2)	0.288
Heavy smoker	19 (6.7)	10 (6.1)	9 (7.6)	0.637

Abbreviations:IQR, interquartile range; VKA, vitamin K antagonists; PPIs, Proton Pump Inhibitors; NSAIDs, non-steroidal anti-inflammatory drugs; WBC white blood count.

Supplementary Table 2 – Validation cohort: comparison of patient outcomes between complicated and uncomplicated diverticulitis

Variable	All population n 282	Uncomplicated diverticulitis n 163	Complicated diverticulitis n 119	p
Death	5 (1.8)	1 (0.6)	4 (3.4)	0.166
Sepsis	10 (3.5)	3 (1.8)	7 (5.8)	0.065
Mechanical ventilation	2 (0.7)	0	2 (1.7)	0.177
Major Complications†	13 (4.6)	5 (3.0)	8 (6.7)	0.148
Any Surgical procedure	27 (9.6)	4 (2.5)	23 (19.3)	<0.001
Major Surgery	20 (7.1)	1 (0.6)	19 (16.0)	<0.001
Percutaneous drainage	7 (2.5)	3 (1.8)	4 (3.4)	<0.001
Colostomy	10 (3.5)	1 (0.6)	9 (7.6)	0.002
LOS ‡	4.6 [1.7 – 8.4]	3.4 [0.4 – 5.3]	6.5 [4.5 – 12.2]	<0.001

Results are expressed as number (percentage) or median (interquartile range) as appropriate.

Abbreviations: LOS, length of hospital stay

†*Major complications include admission to intensive care units/mechanical ventilation, sepsis, or death.*

‡, *LOS is calculated from emergency department admission to hospital discharge.*

Appendix 1

Development of the Predictive Score

Aim of the study was to create a simple score that could accurately predict the risk for complicated AD at patient's bedside evaluation in ED.

Factors associated to complicated AD in our derivation cohort were assessed by univariate analysis (Chi^2 for categorical variables and Mann-Whitney U test for continuous variables).

Factors with significant association to complicated AD were entered into a logistic regression model in order to identify independent predictors of complicated AD in our cohort. Prior to be entered into the logistic models, continuous variables were dichotomized by using ROC analysis. Cut-off for dichotomization was chosen as a value reaching at least 80% specificity for complicated AD in our cohort.

When entered into the logistic regression analysis 6 variables resulted to be independently associated with complicated AD: Sex, Hb, CRP, Obesity, Constipation, absence of PPI therapy. These variables were included in the PACO Score.

To assign a score value to each of the variables, we created a simple linear regression model including all these predictors. The coefficients of these predictors can be understood as the predicted change of complicated diverticulitis risk imparted by each variable by itself, whereas the adjusted coefficients could be intended as the relative contribution of each factor in the cumulative 6 factor model.

Model results were

R = 0.290; R-square = 0.84; Adj R-Square = 0.079.

	**Unstandardized coefficients**			**Standardized coefficients**		
**Variable**	**β**	**Standard error**		**β**	**95% confidence interval**	**P value**
Sex (Male)	0.109	0.026		0.122	0.057 – 0.108	<0.001
Constipation	0.105	0.040		0.076	0.026 – 0.184	0.009
HB <11.9	0.190	0.031		0.183	0.130 – 0.250	<0.001
PCR >80	0.147	0.030		0.142	0.088 – 0.207	<0.001
Obesity	0.305	0.136		0.065	0.038 – 0.573	0.025
Not on PPI	0.109	0.039		0.080	0.031 – 0.186	0.006

Based on standardized coefficients the incremental contribute of each variable to association with complicated diverticulitis was similar (about +10% each). For this reason we assigned 1 point for each Factor

**PACO variables**

Male sex	+ 1 point
PCR >80	+ 1 point
HB <12	+ 1 point
Constipation	+ 1 point
Not on PPI	+ 1 point
Obesity	+ 1 point

After assigning a PACO-diverticulitis score value to each of the patients in derivation and validation cohort we performed a visual calibration of the PACO-diverticulitis score in both cohorts to identify the risk class for each value.

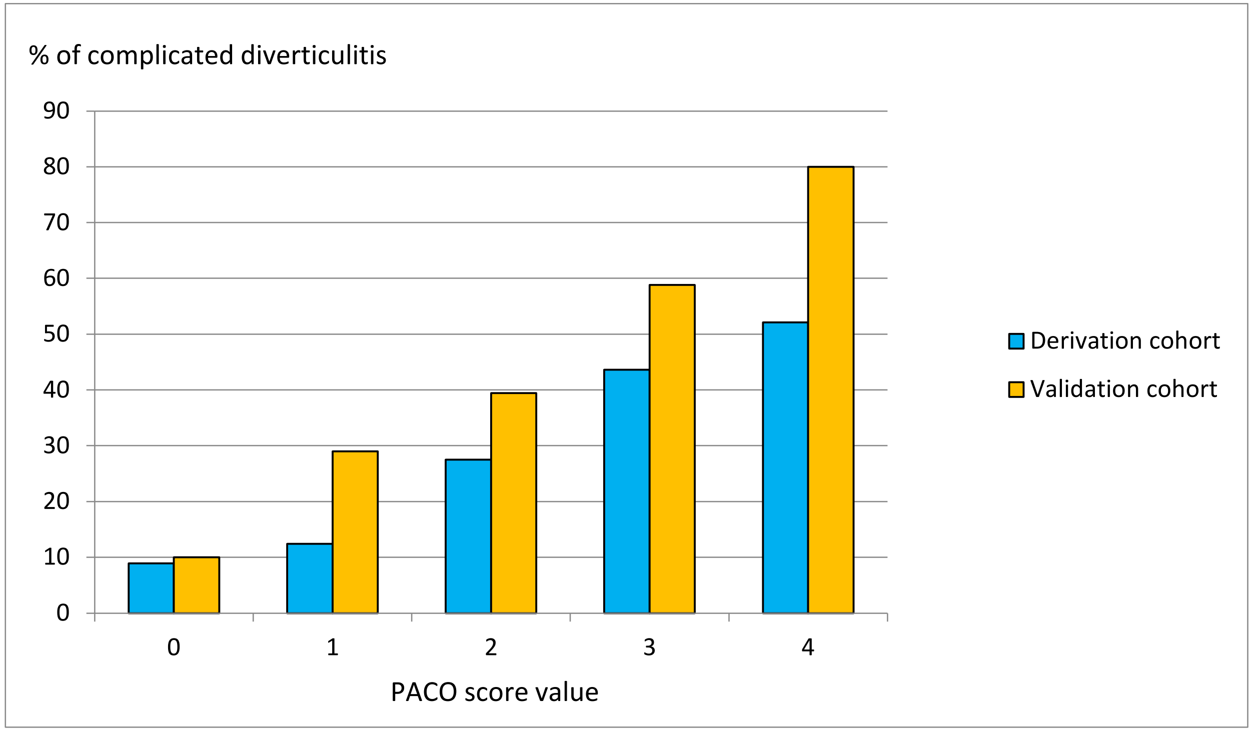

Based on visual calibration data of PACO score in our cohort, we included in low risk group PACO values 0-1, Medium risk group PACO values 2-3 and High risk score PACO ≥ 4
